# Suppurative Thrombophlebitis of the Inferior Vena Cava Resolved with Intravenous Antibiotic Therapy: A Case Report

**Published:** 2018-07

**Authors:** Mahshid Talebi-Taher, Batool Naghavi, Shokoufeh Hajsadeghi, Aida Iranpour, Seyed Mahdi Pahlavani

**Affiliations:** 1 *Rasool-e-Akram General Teaching Hospital, Iran University of Medical Sciences, Tehran, Iran.*; 2 *Rajaie Cardiovascular, Medical, and Research Center, Iran University of Medical Sciences, Tehran, Iran.*; 3 *Department of Internal Medicine, Firoozabadi General Hospital, Iran University of Medical Sciences, Tehran, Iran.*; 4 *Department of Medicine, Beth Israel Deaconess Medical Center, Harvard Medical School, Boston, MA, USA.*

**Keywords:** *Venous thrombosis*, *Pyonephrosis*, *Echocardiography*

## Abstract

Inferior vena cava (IVC) thrombosis is a rare medical condition. Suppurative thrombophlebitis of the IVC is even a more uncommon subtype of IVC thrombosis and is mostly associated with IVC filters or venous catheters. We describe a 66-year-old man with persistent fever and history of pyonephrosis secondary to transurethral lithotripsy 1 month before recent admission. Computed tomography scan of the chest and abdomen revealed a filling defect in the IVC protruding into the right atrium. Transesophageal echocardiogram (TEE) revealed a large mass at the origin of the IVC entering into the right atrium, suggestive of a clot.

Diagnosis of suppurative thrombophlebitis of the IVC secondary to a retroperitoneal abscess was made, and intravenous antibiotic therapy for 6 weeks without anticoagulation conferred ample thrombus resolution. Follow-up TEE in week 16 showed no residual thrombus in the IVC.

## Introduction

Suppurative thrombophlebitis of the inferior vena cava (IVC) is an uncommon presentation form of IVC thrombosis, which is associated with high morbidity and mortality. Its diagnosis is usually based on persistent bacteremia and imaging findings related to thrombosis.^1^^-^^3^

Sepsis could be a predisposing factor for inflammation and venous thrombosis. Bassilios et al.^4^ reported a patient with IVC thrombosis due to acute pyelonephritis that extended to the right atrial cavity, which was confirmed by transesophageal echocardiography (TEE) and successfully treated with IV antibiotics and anticoagulant. 

Most of the previously reported IVC thromboses were secondary to intravenous catheters. Here we present a case of septic thrombosis of the IVC due to a retroperitoneal abscess. 

## Case Report

A 66-year-old man was hospitalized because of persistent fever and chills of 1 month’s duration. He had a history of cholecystectomy, common bile duct anastomosis to the small bowel 5 years previously, and pyonephrosis secondary to transurethral lithotripsy 1 month before the recent admission. He had no history of alcohol consumption and intravenous or illicit drug use.

Positive physical examination findings on the admission day included fever and mild right upper quadrant abdominal tenderness. Initial laboratory test results showed leukocytosis and elevated acute-phase reactants including the erythrocyte sedimentation rate and C reactive protein, while liver enzymes, creatinine, and electrolyte panel were within the normal limits. Three sets of aerobic blood culture, drawn on admission, were negative.

**Figure 1:**
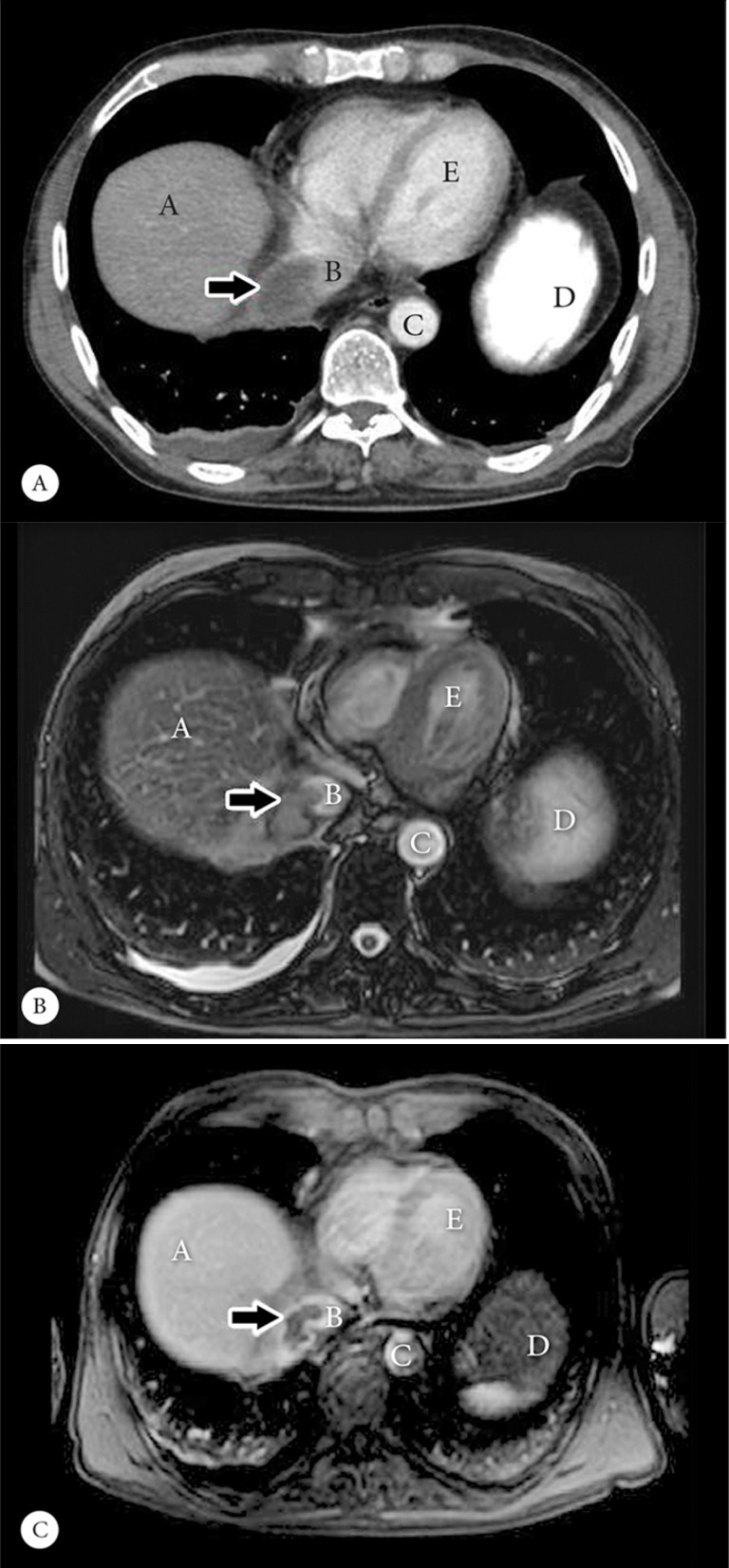
Abdominal contrast-enhanced computed tomographic axial scan (Panel A), showing a non-enhancing filling defect (arrow) in the upper portion of the inferior vena cava with extension to the right atrium. T2 weighted abdominal axial magnetic resonance imaging (MRI) (Panel B), showing a non-enhancing hyposignal filling defect (arrow) that is isosignal to the adjacent liver parenchyma. Contrast-enhanced T1 abdominal MRI with spoiled gradient echo (Panel C), showing a non-enhancing filling defect (arrow) as well as air in the biliary tract, which could be due to previous cholecystectomy and the anastomosis of the common bile duct to the small bowel.

On day 1, computed tomography (CT) scan of the chest and abdomen revealed a filling defect in the IVC, protruding into the right atrium. Fluid accumulation in the retrohepatic and right perinephric space was found. In addition, a hypodense opacity medial to the caudate lobe of the liver, in favor of a hepatic abscess, was noted but not confirmed by the second report (Figure 1). 

**Figure 2:**
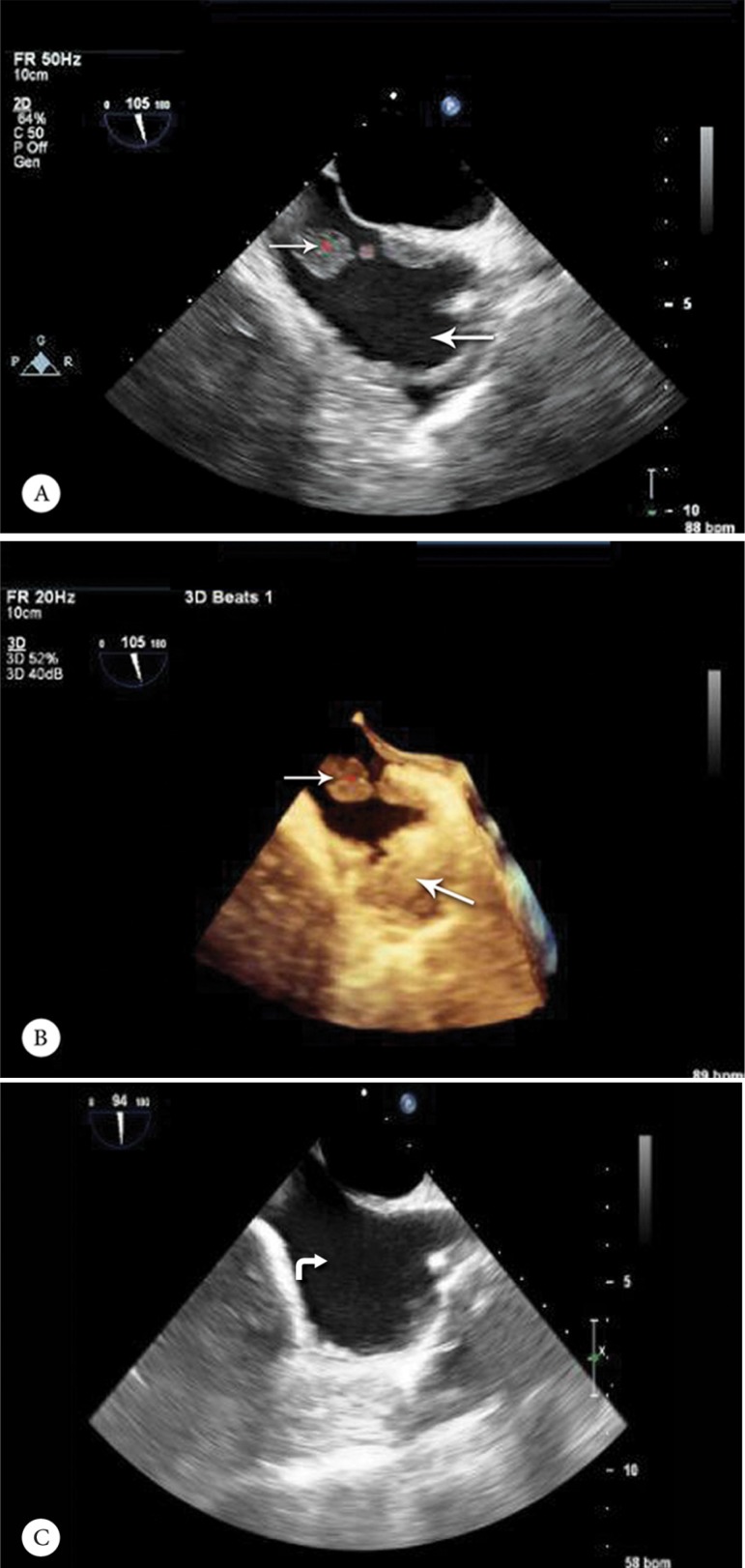
Transesophageal echocardiogram, bicaval view (Panels A and B), showing a large mass (narrow arrow) with 2 fixed (33×12 mm) and mobile (11 mm) components at the origin of the inferior vena cava (IVC) (thick arrow) entering into the right atrium. There was no residual thrombus in the IVC (curved arrow) during week 16 (Panel C).

Transesophageal echocardiography (TEE) revealed interatrial septal aneurysm, small-sized patent foramen ovale with a negligible flow, and no evidence of infective endocarditis. A large mass was detected at the origin of the IVC entering the right atrium, suggestive of a clot (Figure 2).

a, Liver; b, Inferior Vena cava; c, Descending aorta; d, Stomach; e, Heart

**Figure 3:**
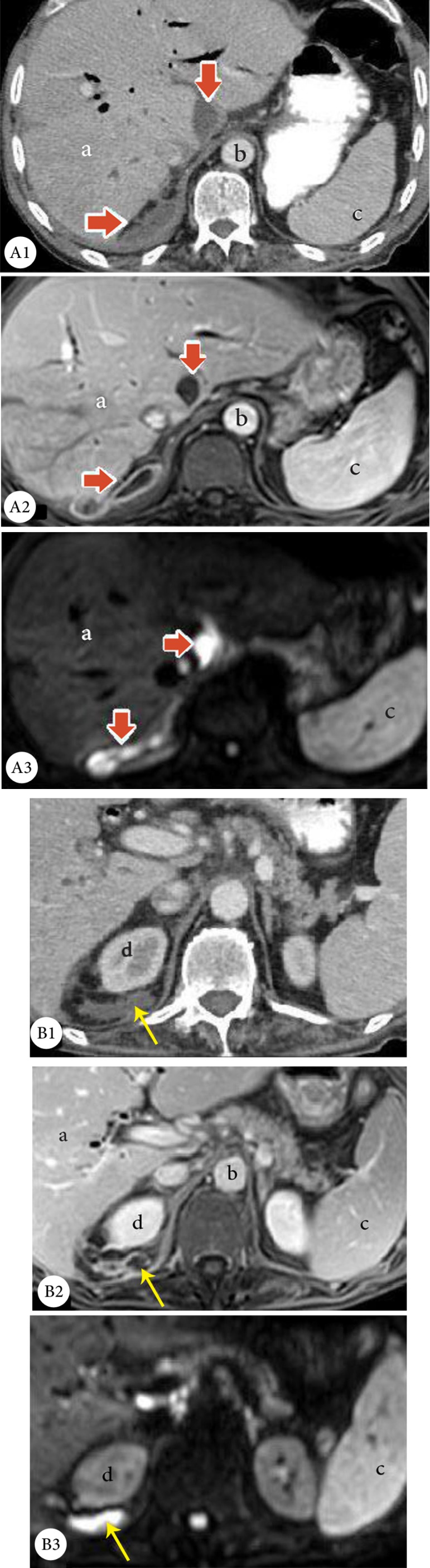
Abdominal contrast-enhanced computerized tomography axial (CT) scan, showing a ring-enhanced collection in the retrohepatic space, medial to the caudate lobe arrows (Panel A1) and the right perinephric space (arrow in Panel B1). Abdominal contrast-enhanced T1 magnetic resonance imaging (MRI), showing the non-central enhancing collection with rim enhancement in the contrast-enhanced T1-spoiled gradient echo image (arrows in Panels A2 and B2). Abdominal diffusion weighted MRI, showing a high signal collection (b-value=1000), in favor of abscess formation (Panels A3 and B3).

Magnetic resonance imaging of the heart, abdomen, and pelvis was performed, and it confirmed the previous findings. It also revealed multiple hyper-intensity signals in the right perinephric space with a restricted pattern and ring enhancement, suggestive of abscess formation (Figure 3).

a, Liver; b, Descending aorta; c, Spleen; d, Right kidney

In light of the imaging and clinical findings, a diagnosis of suppurative thrombosis of the IVC was made and intravenous antibiotic therapy with meropenem (1000 mg every 8 h) in combination with vancomycin (1000 mg every 12 h) was started. This combination was started on suspicion of nosocomial infection bearing in mind that the patient had pyonephrosis secondary to transurethral lithotripsy 1 month before the recent admission. Although experts recommend anticoagulation and endovascular surgery, since our patient’s symptoms had significantly improved after 1 week of antibiotic therapy, we decided to continue the intravenous antibiotics alone as the treatment strategy. Subsequent TEE showed a significant size reduction in the IVC mass. Moreover, a repeat CT scan of the abdomen and pelvis in week 10 showed ample resolution of the perinephric abscesses. Follow-up TEE in week 16 illustrated no residual thrombus in the IVC.

## Discussion

IVC thrombosis is an uncommon medical condition that is associated with significant consequences. It could be classified into 2 main groups according to its etiology^1^:

- Congenital: These thromboses are mostly subclinical and asymptomatic due to extensive collateral development and are caused due to the anatomic anomalies of the IVC.
- Acquired: These thromboses are secondary to a wide range of etiologies such as malignancies, traumas, thrombophilia, infections, and iatrogenic or idiopathic causes.

Suppurative thrombophlebitis of the IVC is an uncommon form of acquired thromboses and usually is associated with the presence of intravenous catheters and IVC filters.^2^

In rare circumstances, suppurative thrombophlebitis could be provoked by propagation from an adjacent infective source, as was the case in our patient. Our patient had a unique clinical scenario: a retroperitoneal abscess formed as a complication of transurethral lithotripsy, which caused IVC invasion and suppurative thrombophlebitis. There are a few case reports in the existing literature where acute pyelonephritis and pyonephrosis were assumed to be predisposing factors for renal vein thrombosis and IVC thrombosis. The authors concluded that renal vein thrombosis and IVC thrombosis could be considered a complication of severe acute pyelonephritis.^4^^, ^^5^

The diagnosis of septic IVC thrombosis is based on persistent bacteremia and imaging findings related to thrombosis.^3^ Although CT scan is a very useful imaging modality for the evaluation of the IVC,^6^ magnetic resonance imaging, and magnetic resonance angiography are more accurate for the measurement of the size and location of IVC thromboses.^7^

Nosocomial organisms such as *Staphylococcus aureus* and *Enterobacteriaceae* are the most common causes of vena cava suppurative thrombophlebitis.^6^ Treatment includes eliminating the source of infection by the administration of intravenous antibiotics with or without surgical intervention and anticoagulation. Surgical excision or ligation is not recommended for vena cava suppurative thrombophlebitis unless the thrombus progresses despite antibiotic and anticoagulation therapy.^8^

There are no controlled studies regarding the treatment of IVC suppurative thrombophlebitis; nonetheless, experts recommend anticoagulation in the setting of vena cava suppurative thrombosis to minimize the risk of pulmonary embolism and prevent post thrombotic sequelae such as pain and edema, especially when there is thrombus extension despite antibiotic therapy.^9^ In our patient, since we observed a substantial clinical response to antibiotic therapy alone, we continued antibiotics for 6 weeks with complete imaging-proven resolution of the thrombosis in addition to clinical improvement.

## Conclusion

The presentation of this case highlights the occurrence of suppurative thrombophlebitis as a complication of retroperitoneal abscesses and ample response to antibiotic therapy without anticoagulation.

## Notes:

***This paper should be cited as: ***Talebi-Taher M, Naghavi B, Hajsadeghi S, Iranpour A, Pahlavani SM. Suppurative Thrombophlebitis of the Inferior Vena Cava Resolved with Intravenous Antibiotic Therapy: A Case Report. J Teh Univ Heart Ctr 2018;13(3):132-135.
